# A chemoenzymatic cascade with the potential to feed the world and allow humans to live in space

**DOI:** 10.1016/j.engmic.2021.100006

**Published:** 2021-11-03

**Authors:** Shuke Wu, Uwe T. Bornscheuer

**Affiliations:** a State Key Laboratory of Agricultural Microbiology, College of Life Science and Technology, Huazhong Agricultural University, Wuhan 430070, China; b Department of Biotechnology & Enzyme Catalysis, Institute of Biochemistry, University Greifswald, D-17487 Greifswald, Germany

**Keywords:** Chemoenzymatic cascade, Cell-free system, Carbon dioxide, Artificial pathway

## Abstract

While the typical targets of (chemo-)enzymatic cascades are fine chemicals (e.g., pharmaceuticals), a chemoenzymatic cascade, artificial starch anabolic pathway (ASAP), was recently developed to synthesize starch from CO_2_. The key results and outstanding features of ASAP are discussed here. We envision that ASAP and its microbial counterpart may enable efficient synthesis of food and sequestration of CO_2_ in a circular manner, thus contributing to a sustainable and hunger-free world and future habitation in space.

A recent trend in biocatalysis research is the transition of traditional single-step reactions into multistep enzymatic cascades (Bell et al., 2021; Yi et al., 2021; Winkler et al., 2021; Sheldon and Woodley, 2018). This transition has been accelerated by the recent tremendous advances in expansion of enzyme toolboxes (Chen and Arnold, 2020), development of computational reaction planning tools (Finnigan et al., 2021), and directed evolution of enzymes (Bornscheuer et al., 2019; Wang et al., 2021). Combining multiple enzymes in one reaction vessel (i.e., *in vitro* system) to form a pathway analogous to that in microbes (i.e., *in vivo* system) enables the streamlined synthesis of the desired final products at higher yields by shifting reaction equilibria and avoiding the tedious problems associated with multistep synthesis (Sperl and Sieber, 2018; Schrittwieser et al., 2018; Kuska and O'Reilly, 2020). Currently, the use of enzyme cascades to produce high value-added fine chemicals is being actively pursued, with the most outstanding examples including the anti-HIV drug Islatravir (Huffman et al., 2019) and the anti-SARS-CoV-2 drug Molnupiravir (McIntosh et al., 2021). These fine chemicals could be rapidly translated into industrial implementation (Wu et al., 2021), which is extremely crucial for emergency applications, such as treating infections caused by SARS-CoV-2. On the other hand, products with much larger volumes have the potential to make a huge difference in human society and on the Earth as a whole. Polymeric materials (plastics) and liquid fuels (e.g., bioethanol) are often the large-volume targets of bioproduction, yet they could be replaced by other materials and energy sources in the future. The main component of food, starch, which is also the main product of agricultural farming, has attracted less attention in industrial biology, probably because it is difficult for bioproduction systems to compete with existing cultivated crops, which have evolved and been under selection for thousands of years. One seminal report, which was published more than seven years ago, describes a four-enzyme cascade for the conversion of cellulose to starch via hydrolysis-polymerization (You et al., 2013), where the raw material, cellulose, is obtained by biosynthesis in plants.

A significant breakthrough was made very recently by Cai et al. who developed a chemo-enzymatic cascade for the synthesis of starch from CO_2_, which is called the artificial starch anabolic pathway (ASAP) (Fig. 1) (Cai et al., 2021). The use of carbon dioxide, which is the hallmark of the third-generation biorefineries, not only frees the process of starch biosynthesis from both fossil fuel-based and plant-based raw materials but also reduces greenhouse gas emissions and enables a circular bioeconomy (Liu et al., 2020; Shi et al., 2020; Intasian et al., 2021). The whole ASAP is divided into four modules: i) a C1 module for conversion of CO_2_ to formaldehyde by a chemical catalyst (ZnO-ZrO_2_) and an alcohol oxidase (AOX); ii) a C3 module for assembling three molecules of formaldehyde into two common C3 metabolites, dihydroxyacetone phosphate (DHAP) and ᴅ-glyceraldehyde-3-phosphate (GAP), by a formolase (FLS), a kinase (DAK), and an isomerase (TPI); iii) a C6 module for combining DHAP with GAP to the key C6 precursor ᴅ-glucose-6-phosphate (G-6-P) by an aldolase (FBA), a phosphatase (FBP), and an isomerase (PGI); and iv) a Cn module accomplishing synthesis of starch via a mutase (PGM), a pyrophosphorylase (AGP), and a starch synthase (SS). Although the whole chemoenzymatic pathway consists of as much as 11 core reactions, it still achieves a rather high product titer (> 1 g L^−1^) and a high productivity (> 300 mg L^−1^ h^−1^). These values are much higher than those achieved with the synthetic crotonyl–coenzyme A (CoA)/ethylmalonyl-CoA/hydroxybutyryl-CoA (CETCH) cycle (Schwander et al., 2016).

**Fig. 1:**
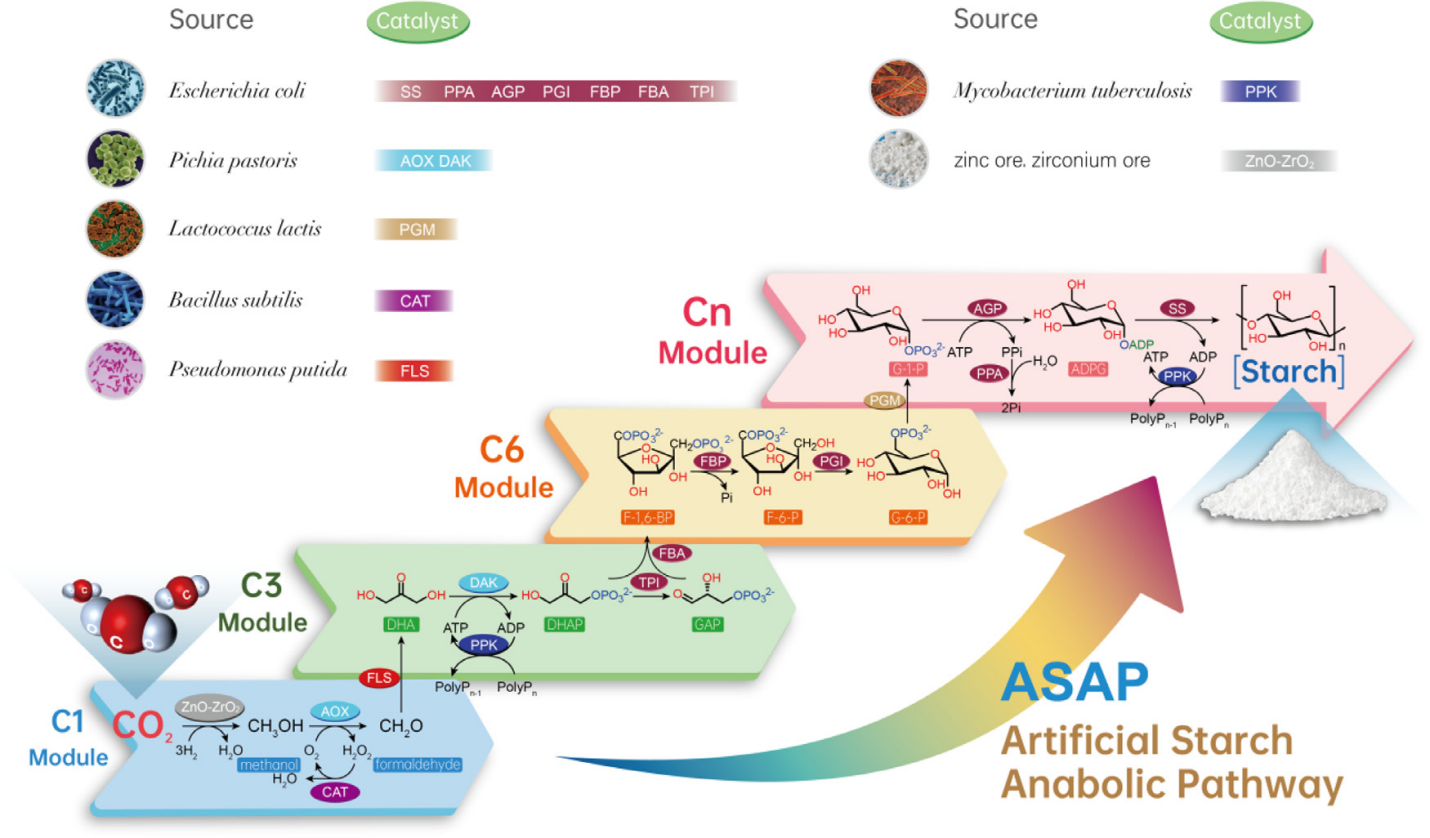
The artificial starch anabolic pathway (ASAP) produces starch from CO_2_. Abbreviations for reaction intermediates: DHA (dihydroxyacetone), DHAP (dihydroxyacetone phosphate), GAP (ᴅ-glyceraldehyde-3-phosphate), F-1,6-BP (ᴅ-fructose-1,6-bisphosphate), F-6-P (ᴅ-fructose-6-phosphate), G-6-P (ᴅ-glucose-6-phosphate), G-1-P (ᴅ-glucose-1-phosphate), ADPG (ADP glucose). Abbreviations for enzymes: AOX (alcohol oxidase), CAT (catalase), FLS (formolase), DAK (dihydroxyacetone kinase), PPK (polyphosphate kinase), TPI (triosephosphate isomerase), FBA (fructose-bisphosphate aldolase), FBP (fructose-bisphosphatase), PGI (phosphoglucose isomerase), PGM (phosphoglucomutase), AGP (ADP-glucose pyrophosphorylase), PPA (pyrophosphatase), SS (starch synthase).

According to our knowledge and experience, the success of ASAP depends on six factors. i) The key C-C bond forming enzymes are available: the crucial enzyme is the formolase (FLS), which was initially computationally designed by Siegel et al. in 2015 (Siegel et al., 2015). FLS converts three molecules of formaldehyde (C1) into dihydroxyacetone (C3). The importance of FLS was also proven as no other alternative enzymes were available for the C3 module. ii) The use of efficient chemical catalyst: for the hydrogenation/reduction of CO_2_, ZnO-ZrO_2_ (Wang et al., 2017), was exploited instead of the enzyme counterpart, formate dehydrogenase, which had only very low CO_2_ reduction activity. This chemoenzymatic cascade combines the best characteristics of chemo- and biocatalysis (Rudroff et al., 2018). iii) Reduction of challenging oxidoreductive reactions: the oxidation state of formaldehyde (CH_2_O) is the same as that of starch (C_6_H_10_O_5_)_n_; therefore, only non-oxidoreductive reactions (phosphorylation, C-C bond formation, and isomerization) are involved in the conversion of formaldehyde to starch. Furthermore, the oxidation of methanol by AOX is highly efficient and does not rely on unstable NAD(P)^+^/NAD(P)H. iv) Selection of the best from several designs: for the C1, C6, and Cn modules, several different designs with alternative enzymes were investigated to choose the best routes. v) Improve bottlenecking enzymes individually: protein engineering of three limiting enzymes (i.e., engineering FLS and AGP for improved activity and FBP for reduced inhibition) drastically boosted the efficiency of the whole pathway. vi) Optimization of the whole process: temporal and spatial separation of the chemoenzymatic process allows the chemo- and biocatalysts to work under optimal conditions, and this further enhances the efficiency of the whole process. The final chemoenzymatic ASAP outperformed maize (a C4 plant) in terms of both the starch synthesis rate and the theoretical solar-to-starch efficiency, demonstrating that human-designed, laboratory-engineered cell-free biocatalytic systems can outcompete nature-evolved organisms.

In the hallmark publication by Cai et al. (Cai et al., 2021), the use of ASAP was successfully demonstrated *in vitro*. Although a cell-free system can achieve high product yields and titers by obviating problems with living cells, it suffers from high enzyme production costs and stability issues (Bowie et al., 2020). The *in vivo* approach, i.e., engineering microbes, may provide an alternative method for practical applications; for example, the metabolic engineering of microbes for industrial manufacturing of commodity chemicals, such as 1,4-butanediol (Yim et al., 2011) and eicosapentaenoic acid (Xue et al., 2013), has been well demonstrated. In our opinion, the advantage of life science over chemistry is self-replication via autotrophic or heterotrophic mechanisms. The enzymes in microbes can always be regenerated, whereas the enzymes in test tubes will always lose activity. However, there are often unavoidable problems when engineering heterogenous or artificial pathways in microbes, such as toxicity of substrates/intermediates/products and the trade-off between production and cell growth, which prevent high productivity and titers from being achieved. In the case of the conversion of methanol to starch, fortunately, many natural methylotrophic microbes (e.g., *Pichia*) are easily available, genetically engineerable, and naturally resistant to methanol/formaldehyde (Cotton et al., 2020; Gao and Zhou, 2020). Thus, they are promising hosts for engineering starch production from methanol via ASAP or other native/artificial pathways. Chemical catalysts could be coupled with these engineered methylotrophic microbes to achieve the conversion of CO_2_ (and H_2_) to starch. In addition to methylotrophic microbes, natural chemoautotrophic microbes (e.g., acetogens) could also be interesting hosts for this application because they can fix CO_2_ using H_2_ (or other electron donors) via natural pathways (e.g., the Wood-Ljungdahl pathway) (Claassens et al., 2016; Jiang et al., 2021). The use of chemoautotrophic microbes will eliminate the use of chemical catalysts for the initial hydrogenation of CO_2_ and enable a purely enzymatic one-pot transformation of CO_2_ to produce starch. It will be exciting to see the use of engineered microbes in parallel with the *in vitro* ASAP pathway.

Because the substrate, CO_2_, is the major greenhouse gas, while the product, starch, is the major component of food, the ASAP provides an exciting opportunity to address two great challenges at the same time: climate change and global hunger (Prather, 2020). We envision that there are enormous opportunities to engineer microbes (especially methylotrophs and chemoautotrophs) to achieve similar transformations of CO_2_ to food or other commodity chemicals. All these efforts and developments in the field of biomanufacturing, both *in vitro* and *in vivo*, will contribute to shaping a sustainable and hunger-free world. What is even more exciting is the circular nature of the conversion of CO_2_ to starch (by ASAP) and starch to CO_2_ (by humans or other heterotrophs); thus, the ASAP and its microbial counterpart may be very promising strategies for future travel and habitation in Space.

## Declaration of competing interest

The authors declare that they have no known competing financial interests or personal relationships that could have appeared to influence the work reported in this paper.
